# The effects of ERCC1 expression levels on the chemosensitivity of gastric cancer cells to platinum agents and survival in gastric cancer patients treated with oxaliplatin-based adjuvant chemotherapy

**DOI:** 10.3892/ol.2012.1096

**Published:** 2012-12-28

**Authors:** YONG-PING LIU, YANG LING, QIU-FENG QI, YA-PING ZHANG, CHANG-SONG ZHANG, CHANG-TAI ZHU, MEI-HUA WANG, YAO-DONG PAN

**Affiliations:** 1 Clinical Oncology Laboratory; Changzhou Tumor Hospital Affiliated to Suzhou University, Changzhou 213002, P.R. China; 2 Department of Pathology; Changzhou Tumor Hospital Affiliated to Suzhou University, Changzhou 213002, P.R. China; 3 Department of Gastroenterological Surgery, Changzhou Tumor Hospital Affiliated to Suzhou University, Changzhou 213002, P.R. China

**Keywords:** excision repair cross-complementing gene 1, gastric cancer, chemosensitivity, adjuvant chemotherapy, platinum, prognostic factor

## Abstract

Excision repair cross-complementing 1 (ERCC1) is reported to be involved in the sensitivity of cancer cells to platinum-based chemotherapy. The present study was designed to evaluate the effects of ERCC1 expression on the chemosensitivity of platinum agents in gastric cancer cell lines, and on survival in gastric cancer patients treated with surgery followed by oxaliplatin-based adjuvant chemotherapy. ERCC1 expression levels were measured by quantitative reverse transcription-polymerase chain reaction (qRT-PCR) and western blot analysis, respectively. The chemosensitivity of a series of gastric cancer cell lines to platinum agents *in vitro* was evaluated using CellTiter 96 Aqueous One Solution Cell Proliferation Assay kit. The apoptotic effect of the drugs was evaluated by double staining with Annexin-V-fluorescein isothiocyanate (FITC) and propidium iodide (PI). The results demonstrated that the expression levels of ERCC1 mRNA were correlated with the chemosensitivity of platinum agents, and depletion of ERCC1 sensitized the relatively resistant MKN45 cells to cisplatin and oxaliplatin. Univariate analyses revealed that patients with low ERCC1 levels had longer relapse-free survival (RFS) and overall survival (OS) than those with high ERCC1 levels (median RFS, 18 vs. 7 months, P=0.001; median OS, 27 vs. 11 months, P=0.001). Multivariate analyses suggested that high ERCC1 expression is an independent prognostic marker of poor RFS [hazard ratio (HR), 2.16; 95% confidence interval (CI), 1.09–4.25; P= 0.026] and OS (HR, 2.21; 95% CI, 1.07–4.55; P=0.031). These results suggest that overexpression of ERCC1 is correlated with platinum drug resistance in gastric cancer cells, and that depletion of ERCC1 sensitizes gastric cancer cell lines to cisplatin and oxaliplatin. Gastric cancer patients with low levels of ERCC1 expression demonstrate a benefit from oxaliplatin-based adjuvant chemotherapy.

## Introduction

Despite its declining incidence, gastric cancer remains the second most common cause of cancer-related mortality in Asia and worldwide (1,2). Surgery remains the mainstay of any curative treatment. However, even following radical surgery, the majority of gastric cancer patients develop local or distant recurrence (3). Several meta-analyses of postoperative adjuvant trials have demonstrated a significant benefit for chemotherapy-treated patients (4). However, certain patients have undergone expensive and potentially harmful therapy without gaining any benefit. Thus, the identification of molecular markers in resected tumor tissues that are able to predict outcomes is essential for the future development of adjuvant chemotherapy for gastric cancer patients.

Cisplatin is widely used and has been demonstrated to be effective in the palliative treatment of gastric cancer, and different combinations have been investigated in the adjuvant setting (5,6). Oxaliplatin (cis-[oxalate (trans-l-1, 2-diaminocyclohexane) platinum (II)]), a third-generation platinum compound, has been observed to be more effective than cisplatin (7), and has a more favorable toxicity profile than cisplatin (8). Thus the oxaliplatin plus 5-fluorouracil (5-FU) modulated with leucovorin (LV) (FOLFOX regimen) has been widely used as the first-line treatment in advanced gastric cancer (9–13). However, resistance to oxaliplatin and cisplatin remains a major obstacle to further increasing the response rate. Additionally, the involvement of the FOLFOX regimen in combination with surgery to increase local control or prolong survival also requires further investigation in resected gastric cancer.

DNA repair capacity is considered to be both a barrier to carcinogenesis and a crucial molecular pathway implicated in resistance to platinum-based chemotherapy (14). The cytotoxic effect of anticancer platinum drugs is principally attributable to the formation of platinum-DNA adducts (15). Nucleotide excision repair (NER) is the primary DNA repair mechanism that removes platinum-DNA adducts from genomic DNA. Excision repair cross-complementing 1 (ERCC1) is a critical gene in the NER pathway (16). ERCC1 is highly conserved during evolution and is constitutively expressed in all tissues at relatively high levels. A functional ERCC1 is essential for survival; knockdown of the ERCC1 gene in mice was observed to lead to an accelerated-aging phenotype, with brain damage, liver failure and death occurring shortly after weaning (17). A growing body of evidence has demonstrated that the ERCC1 gene acts as both a predictive and a prognostic marker (18,19). It has been demonstrated in several clinical studies that ERCC1 messenger RNA expression in various types of cancer, including ovarian, colorectal, gastric and esophageal cancer, as well as non-small cell lung cancer (NSCLC), is correlated with clinical resistance to platinum agents (20–23). However, a limited number of gastric cancer studies have focused on the effect of ERCC1 expression on the outcome of FOLFOX adjuvant chemotherapy.

The aim of this study was to evaluate the effect of ERCC1 expression levels on the chemosensitivity of platinum agents in gastric cancer cell lines, and to evaluate whether ERCC1 expression levels are correlated with survival in gastric cancer patients treated with surgery followed by oxaliplatin-based adjuvant chemotherapy.

## Materials and methods

### Cell lines and cultures

The human gastric cancer cell lines, including AGS, NCI-N87, BGC-823, HGC and MKN45, were obtained from the Shanghai Institute of Cell Biology (Shanghai, China). All cell lines were propagated in Roswell Park Memorial Institute (RPMI)-1640 medium (Gibco-BRL; Carlsbad, CA, USA), supplemented with 10% bovine serum, penicillin (100 U/ml)-streptomycin (100 mg/ml), pyruvate, glutamine and insulin at 37°C in a water-saturated atmosphere with 5% CO_2_.

### Drugs

Oxaliplatin (Oxa) and cisplatin (DDP) were supplied by the Jiangsu Hengrui Medicine Company (Jiangsu, China). The dilutions of all reagents were freshly prepared before each experiment. CellTiter 96 Aqueous One Solution Cell Proliferation Assay kit was purchased from Promega Corporation (Madison, WI, USA). Annexin-V-fluorescein isothiocyanate (FITC) Apoptosis Detection kit was purchased from Invitrogen Life Technologies (Carlsbad, CA, USA).

### siRNA-mediated ERCC1 silencing

Transient knockdown of ERCC1 was achieved by transient transfection of 10 ng/*μ*l ERCC1 siRNA (OriGene Technologies, Inc.; Rockville, MD, USA). AGS and MKN cells were transfected with siRNA duplexes (10 ng/*μ*l) using Lipofectamine 2000 (Invitrogen Life Technologies) for 48 h, according to the manufacturer’s instructions, and then treated with platinum agents for 48 h. An empty pGFP-V-RS vector and HuSH 29-mer non-effective scrambled pGFP-V-RS were used in control experiments, and were purchased from OriGene Technologies, Inc.

### Cell viability assay

Cytotoxicity was determined using the CellTiter 96 Aqueous One Solution Cell Proliferation Assay kit. Briefly, tumor cells growing in the log-phase were trypsinized and seeded at 2×10^3^ cells/well into 96-well plates and allowed to attach overnight. The medium in each well was replaced with fresh medium or medium with various concentrations of drugs in at least six replicate wells and left to make contact for 48 h. One-fifth of the volume of CellTiter 96 Aqueous One Solution was added to each well and incubated for an additional 3 h. Absorbance was determined with a microplate reader (Bio-Rad; Hercules, CA, USA) at 490 nm. The blank control wells were used for zeroing the absorbance. Each experiment was allocated 10 wells containing drug-free medium as a control. The inhibition rate (I%) was calculated using the background-corrected absorbance by the following equation: I%=(A_untreated control well_ - A_experimental well_) / A_untreated control well_ ×100. The IC_50_ value was defined as the concentration required for 50% inhibition of cell growth.

### Apoptosis assay

The quantification of apoptotic cells was performed using an Annexin-V-FITC Apoptosis Detection kit (Invitrogen Life Technologies) according to the manufacturer’s instructions. Briefly, cells were plated in a 60-mm Petri dish and allowed to grow to 75–80% confluence. Cells were exposed to ERCC1 siRNA and anticancer drugs were added singly for 48 h or cells were pretreated with ERCC1 siRNA for 48 h. Subsequently, the medium was replaced with fresh medium with anticancer drugs for another 48 h, and these were compared with control cells that had not been treated with drugs. The cells were then collected and resuspended in 500 *μ*l binding buffer, and 5 *μ*l Annexin-V-FITC and 5 *μ*l propidium iodide (PI) were added. Analyses were performed with a flow cytometer (FACSCalibur; Becton Dickinson; Franklin Lakes, NJ, USA).

### Quantitative polymerase chain reaction (qPCR) assessment of ERCC1 expression

Fresh specimens were collected, grossly viewed and dissected from the primary malignant lesion by a pathologist immediately after surgical resection, and frozen within 20 min in liquid nitrogen. Cells were harvested with trypsin, washed with PBS and collected by centrifugation at 1,000 rpm for 5 min. Total RNA was extracted using SV Total RNA isolation system (Promega Corporation) according to the manufacturer’s instructions. The purity and quality of the mRNA were measured by a Bio-visible spectrophotometer (Eppendorf AG; Hamburg, Germany), while 1% agarose gel electrophoresis was used to assess the integrity of the obtained RNA. cDNA with a total volume of 20 *μ*l was synthesized using the reverse transcription system containing reverse transcriptase (Promega Corporation) according to the manufacturer’s instructions. Real-time qPCR of the target gene and β-actin as internal control was carried out with iCycler iQ Multicolor Real-time PCR Detection System (Bio-Rad). The 20 *μ*l PCR reaction mixture contained 1X primers and probe mixture [assay IDs: Hs00157415_m1 (ERCC1) and Hs99999903_m1 (β-actin); Applied Biosystems, Foster City, CA, USA] and 1X Absolute QPCR mix (Abgene UK, Ltd.; Surrey, UK). The PCR conditions were 50°C for 2 min, 95°C for 15 min, followed by 45 cycles at 95°C for 15 sec and 60°C for 1 min. Relative gene expression quantifications were calculated according to the comparative Ct method using β-actin as an endogenous control and commercial human total RNA (Clontech Laboratories, Inc.; Mountain View, CA, USA) as calibrators. Final results were determined by the 2^−ΔΔCt^ formula, as described previously by Livak and Schmittgen (24). In the siRNA-mediated ERCC1 silencing study, equal amounts of the qRT-PCR products were also analyzed in ethidium bromide-stained 1% agarose gel.

### Western blot analysis

ERCC1 protein expression in cells was detected by western blot analysis. Briefly, cells were washed twice with ice-cold phosphate-buffered saline (PBS). Total protein lysates were obtained from cells using radio immuno-precipitation assay (RIPA) cell lysis buffer (Boster, Wuhan, China). Samples were spun at 20,000 x g at −4°C for 15 min, and the supernatant was stored at −80°C or immediately quantified using a protein assay (Bio-Rad). Protein lysates were electrophoresed and equal loading was assessed by Ponceau Red staining following transfer to nitrocellulose membranes. The primary antibodies used for blotting were anti-ERCC1 and anti-β-actin (OriGene Technologies, Inc.) as a loading control. The secondary antibody used was goat anti-mouse-horseradish peroxidase (HRP) (Santa Cruz Biotechnology, Inc.; Santa Cruz, CA, USA). Luminescence was revealed by incubation with ECL Western blotting substrate (Promega Corporation) and signals were detected using a FluorChem SP imaging system (Alpha Innotech, San Leandro, CA, USA).

### Patients and treatment protocols

Tumor specimens were collected from 75 patients with stages Ib-IV (M0) who were recruited during the period from January, 2005 to June, 2007 and underwent surgery at the Department of Gastroenterological Surgery, Changzhou Tumor Hospital. The patients comprised 53 males and 22 females, ranging in age from 36–73 years, with a median age of 58 years. None of the patients had previously received chemotherapy. This study had been approved by the local ethics committees, and written informed consent was obtained from all patients. Following surgery, 57 patients received ≥6 cycles of oxaliplatin at 85 mg/m^2^ plus leucovorin at 20 mg/m^2^ on the first day of treatment, followed by 5-FU, via a 400 mg/m^2^ bolus, and a 22 h continuous infusion of 600 mg/m^2^ 5-FU on Days 1–2 at 2-week intervals. Twenty-three patients received surgery alone.

### Follow-up

Interim history, physical examination, hematological studies, carcinoembryonic antigen levels and whole-body computed tomography were conducted every 3 months in the first year and every 6 months thereafter. Patients underwent upper endoscopy 6 months after surgery and every 12 months thereafter. Recurrences or metastases of gastric carcinoma were determined by cytology biopsy, surgery or whole-body computed tomography. The American Joint Committee on Cancer (AJCC) 7th Edition of Gastric Cancer was used for the classification of each case. The study was implemented in a blind fashion; the patient outcome was unknown to investigators performing the molecular analyses. Relapse-free survival (RFS) was the time period from study initiation until disease recurrence or death or the day of the last follow-up visit (whichever of these occurred first). Overall survival (OS) was the time period from study initiation until the date of mortality, regardless of the cause, or until the most recently documented follow-up.

### Statistical analysis

Statistical significance was based on a two-sided significance level of 0.05. All analyses were performed with the Statistical Package for the Social Sciences (SPSS), version 13.0 (SPSS, Inc.; Chicago, IL, USA). The Spearman’s correlation coefficient was adopted to analyze the correlation between gene expression levels and drug sensitivity. Statistical comparisons were performed using the Student’s t-test. To test for correlations between gene expression levels and the clinical variables, dichotomization of the gene expression values as equal/above and below the median expression value were conducted and tested by the χ^2^ test or Fisher’s exact test (two-sided), as appropriate. Kaplan-Meier survival curves and the log-rank test were used to analyze univariate distributions for RFS and OS. The prognostic significance of ERCC1 following adjustment for other prognostic factors was assessed using a Cox proportional hazards regression model.

## Results

### ERCC1 expression level is correlated with the chemosensitivity of platinum agents in gastric cancer cell lines

**Figure 1:**
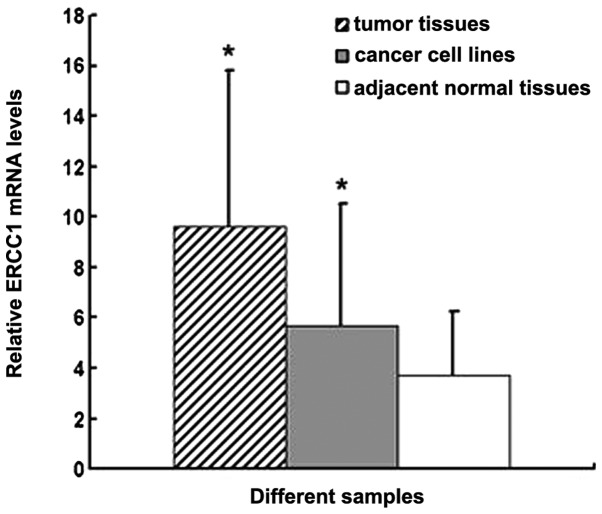
Relative excision repair cross-complementing 1 (ERCC1) mRNA levels among gastric cancer tissues, cancer cell lines and adjacent normal tissues. ERCC1 mRNA was more highly expressed in cancer tissues and cancer cell lines compared with adjacent normal tissues (^*^P<0.05).

The ERCC1 expression levels were first examined in 7 gastric cancer cell lines, tumor tissues and adjacent normal tissues by qRT-PCR. ERCC1 mRNA levels in gastric cancer cell lines and gastric cancer tissues were significantly higher than those in adjacent normal tissues (P<0.05; Fig. 1). No significant differences were observed between gastric cancer cell lines and gastric cancer tissues.

**Figure 2:**
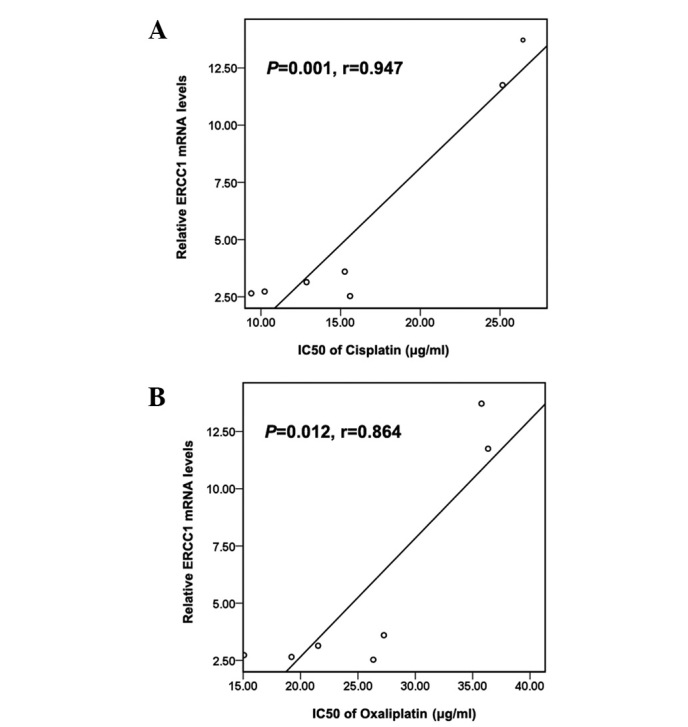
Excision repair cross-complementing 1 (ERCC1) expression levels are correlated with the chemosensitivity of platinum agents in gastric cancer cell lines. (A) ERCC1 mRNA expression levels are positively correlated with the IC_50_ value of cisplatin in 7 gastric cancer cell lines (P=0.001; r=0.947). (B) ERCC1 mRNA expression levels are positively correlated with the IC_50_ value of oxaliplatin in 7 gastric cancer cell lines (P=0.012; r=0.864).

The correlation between ERCC1 mRNA expression levels and the chemosensitivity of platinum agents in 7 gastric cancer cell lines was subsequently determined. We found that ERCC1 mRNA expression levels were positively correlated with the IC_50_ value of cisplatin (P=0.001; r=0.947; Fig. 2A) and oxaliplatin (P=0.012; r=0.864; Fig. 2B), respectively.

### Inhibition of ERCC1 by siRNA sensitizes gastric cancer cell lines to cisplatin and oxaliplatin

**Figure 3:**
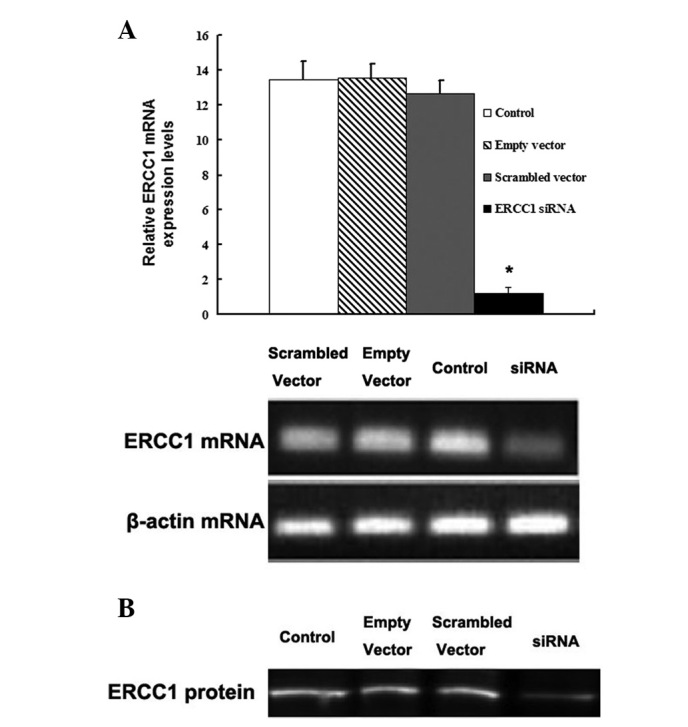
Effect of siRNA on excision repair cross-complementing 1 (ERCC1) mRNA and protein expression in the MKN45 cells. (A) ERCC1 siRNA significantly downregulated the expression level of ERCC1 mRNA in MKN45 cells (^*^P<0.05, compared with the untreated control group). (B) ERCC1 siRNA significantly downregulated the expression level of ERCC1 protein in MKN45 cells.

**Figure 4:**
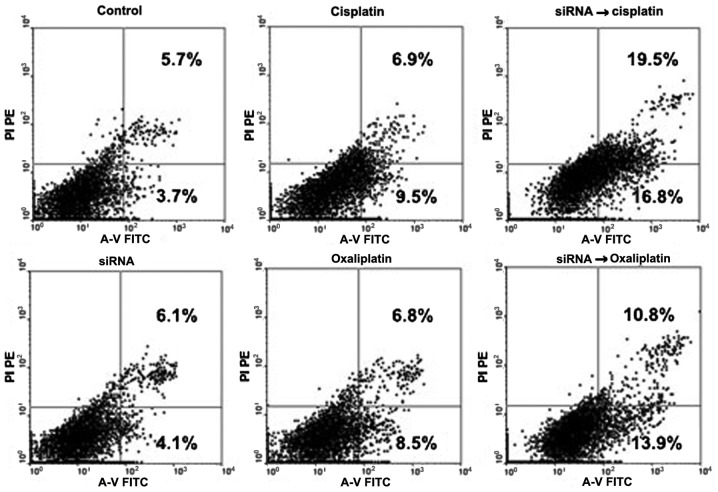
Effect of siRNA-mediated downregulation of excision repair cross-complementing 1 (ERCC1) on early apoptosis rates in relatively resistant MKN45 cell line. The cells were treated with ERCC1 siRNA (10 ng/*μ*l) and platinum agents (IC20) alone for 48 h, or transfected with 10 ng/*μ*l ERCC1 siRNA for 48 h, then followed by platinum agents for another 48 h. The apoptotic effect of the drugs was evaluated by double staining with both Annexin-V-fluorescein isothiocyanate (FITC) and propidium iodide (PI).

To further examine the functional role of ERCC1 in gastric cancer cells, the relatively resistant MKN45 cells were transfected with siRNA duplexes against ERCC1. In MKN45 cell lines, successful knockdown of ERCC1 expression was confirmed by qRT-PCR and western blot analysis (Fig. 3). Downregulation of ERCC1 by siRNA did not result in significant suppression of cell proliferation following transfection for 48 h, while siRNA-mediated attenuation of ERCC1 expression led to a subsequent sensitizing effect to cisplatin and oxaliplatin by an early apoptosis test (Fig. 4). The CellTiter 96 Aqueous One Solution Cell Proliferation test demonstrated that downregulation of ERCC1 by siRNA decreased the IC_50_ value of cisplatin from 26.44±2.72 to 2.12±0.31 *μ*g/ml (P<0.001) and that of oxaliplatin from 35.77±3.82 to 7.12±0.72 *μ*g/ml (P=0.003). These results suggest that the ERCC1 expression level is important for cell viability against platinum-based drugs, and that the expression of ERCC1 siRNA effectively increased the sensitivity to these drugs.

### Expression levels of ERCC1 mRNA are correlated with clinicopathological characteristics

The expression of ERCC1 mRNA was evaluable in all 75 patients, and the median value was 7.32 (range 0.50–147.03). Table I summarizes the characteristics of the study population. Patients were divided into two groups centered about the median value; 38 patients with high ERCC1 levels and 37 with low ERCC1 levels. High ERCC1 expression was more common in younger patients (60.5% for younger patients vs. 37.8% for elderly patients; P=0.049). No other correlations were observed between the clinical characteristics and ERCC1 expression levels (Table I).

### Expression levels of ERCC1 mRNA are correlated with survival in patients receiving surgery followed by FOLFOX adjuvant chemotherapy

**Figure 5:**
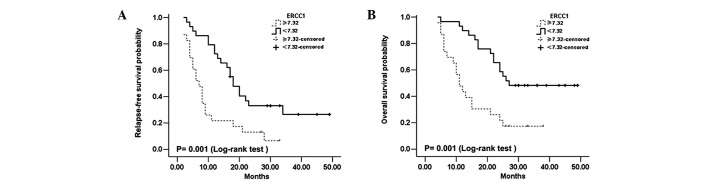
Relapse-free survival curve (A) and overall survival curve (B) in patients treated with oxaliplatin-based adjuvant chemotherapy, according to excision repair cross-complementing 1 (ERCC1) expression equal/above and below the median ERCC1 level. Patients with ERCC1 levels below the median had significantly longer median RFS and OS times compared with patients with ERCC1 levels equal/above the median (median RFS, 18 vs. 7 months; median OS, 27 vs. 11 months, respectively).

The median RFS was 12.5 months (range, 2–49 months), and the median OS time was 22 months (range, 4–49 months). Table II demonstrates that ERCC1 expression is significantly correlated with both RFS (P= 0.001) and OS (P=0.001) time, Fig. 5A and B show the Kaplan-Meier survival curve for patients with intratumoral ERCC1 levels equal/above and below the median ERCC1 level. Patients with ERCC1 levels below the median had a significantly longer median RFS and median OS times compared with patients with ERCC1 levels equal/above the median (median RFS, 18 vs. 7 months; median OS, 27 vs. 11 months), respectively. Other factors that were significantly correlated with RFS and OS in the univariate analysis by the Kaplan-Meier survival curves and the log-rank test included age, tumor stage and the levels of serum carcinoembryonic antigen (Table II). Gender, tumor differentiation and tumor location were not significant prognostic factors for either RFS and OS. ERCC1 levels, stage and serum carcinoembryonic antigen remained significant prognostic factors correlated with RFS and OS in the Cox proportional hazards regression model multivariate analysis (Table III).

## Discussion

Platinum-based chemotherapy remains the backbone of therapy in the management of advanced gastric cancer. Recently, oxaliplatin, a third platinum analog, has been widely used in patients with gastric cancer (25). A small number of studies have demonstrated that the combination of oxaliplatin and 5-FU modulated with LV obtained an objective response rate of 38–55% in gastric cancer patients (9–13,26). However, this implies that ∼50% of patients suffered the toxic effects of this regimen without obtaining any real benefit. Therefore, predictive markers are required to identify those patients likely to benefit from oxaliplatin-based treatment in gastric cancer.

The cytotoxic effects of cisplatin and oxaliplatin are principally attributable to the formation of bulky platinum-DNA adducts (7,27), and these adducts are recognized and repaired by the nucleotide excision repair (NER) pathway. The ERCC1 protein is major component of the NER complex, acting as the rate-limiting enzyme in the NER pathway, while high expression of ERCC1 has been demonstrated to be correlated with poor responses to chemotherapy in various tumor types (19,28–33).

In our study, we found the ERCC1 expression levels were inversely associated with the chemosensitivity of platinum agents in 7 gastric cancer cell lines, and the inhibition of ERCC1 expression by siRNA sensitized the effects of cisplatin and oxaliplatin in the relatively resistant MKN45 cells. These results were partially consistent with those of other studies (28,34,35). The mechanisms whereby ERCC1 participates in platinum resistance in cancer cells has been demonstrated to be correlated with increased removal of the platinum-DNA adducts and interstrand cross-links (36–38).

Several studies have investigated the influence of ERCC1 in resistance to platinum compound in gastric cancer patients, and the majority of which revealed that patients with low levels of ERCC1 protein or mRNA expression were associated with favorable clinical outcomes of platinum based anti-cancer chemotherapy (32,39,40). This suggests that ERCC1 is a predictive marker for clinical resistance to platinum compounds. Our results demonstrated that patients with low ERCC1 levels had longer RFS and OS times than those with high ERCC1 levels, and the multivariate analysis suggested that ERCC1 expression is an independent predictive marker associated with RFS and OS, which is consistent with the studies mentioned previously. By contrast, other studies have demonstrated that low ERCC1 expression was correlated with poor survival (41) or exhibited no correlation with survival (42). Conflicting results between different studies may be related to biological variations of the analyzed tumors, or to variations with respect to the chemotherapeutic protocol or to the different techniques for testing ERCC1 expression.

We also investigated the correlation between ERCC1 expression levels and clinicopathological characteristics. A significant correlation was only observed between ERCC1 expression levels and age (P=0.049), and high ERCC1 expression was more common in younger patients (60.5% for younger patients vs. 37.8% for elderly patients), which may explain why younger patients had poorer RFS and OS times than elderly patients, following oxaliplatin-based adjuvant chemotherapy (median RFS, 8 vs. 18 months, P=0.017; median OS, 15 months vs. undefined, P=0.019, respectively; Table II).

One limitation of the present study is the relatively small sample size. Moreover, the majority of patients who received surgery alone belonged to stages II and III, but were not willing to receive the adjuvant chemotherapy or radiotherapy. Due to the imbalance in the distribution of clinical stage and the inadequate sample size, we did not compare survival times between patients receiving adjuvant chemotherapy and those treated with surgery alone.

In conclusion, the present results support the theories that ERCC1 may participate in platinum resistance in gastric cancer cells, and that high ERCC1 expression may be a poor predictor of efficient oxaliplatin-based adjuvant chemotherapy. To further confirm the prognostic value of tumor ERCC1 expression in gastric cancer, a multi-center prospective study with a large sample size is required in our future investigations.

## Figures and Tables

**Table I:** Correlations between ERCC1 expression levels and clinical variables.

Variables	ERCC1 expression level		Total	P-value
	High (%)	Low (%)		
Gender				0.276
Male	24 (45.3)	29 (54.7)	53 (100)	
Female	13 (59.1)	9 (40.9)	22 (100)	
Age (years) (median)				0.049
≤58	23 (60.5)	15 (39.5%)	38 (100)	
>58	14 (37.8)	23 (62.2%)	37 (100)	
Tumor differentiation				0.537
Well	15 (57.7)	11 (42.3)	26 (100)	
Moderate	17 (43.6)	22 (56.4)	39 (100)	
Poor or undifferentiated	5 (50.0)	5 (50.0)	10 (100)	
Site of tumor				0.427
Proximal stomach	12 (50.0)	12 (50.0)	24 (100)	
Stomach body	10 (62.5)	6 (37.5)	16 (100)	
Distal stomach	15 (42.9)	20 (57.1)	35 (100)	
Staging				0.311
I and II	9 (56.3)	7 (43.7)	16 (100)	
III	13 (39.4)	20 (60.6)	33 (100)	
IV	15 (57.7)	11 (42.3)	26 (100)	
Carcinoembryonic antigen (ng/ml)				0.296
≤5	21 (44.7)	26 (55.3)	47 (100)	
>5	16 (57.1)	12 (42.9)	28 (100)	

ERCC1, excision repair cross-complementation group 1.

**Table II:** Factors correlated with survival in patients receiving surgery followed by oxaliplatin-based adjuvant chemotherapy.

	n	M-RFS (months)	P-value	MST (months)	P-value
Gender			0.526		0.408
Male	37	14		24	
Female	15	9		21	
Age (years)			0.017		0.019
≤58	25	8		15	
>58	27	18			
Tumor differentiation			0.652		0.419
Well	20	17		22	
Moderate	25	12		22	
Undifferentiated	7	8		15	
Tumor location			0.484		0.598
Proximal stomach	14	10		17	
Stomach body	10	13		21	
Distal stomach	28	16		24	
Staging			<0.001		<0.001
I, II and III	32	18			
IV	20	6		11	
ERCC1 level			0.001		0.001
<7.32	29	18		27	
≥7.32	23	7		12	
Carcinoembryonic antigen (ng/ml)			<0.001		<0.001
≤5	33	18		27	
>5	19	6		12	

ERCC1, excision repair cross-complementation group 1; M-RFS, median relapse-free survival; MST, median survival time.

**Table III:** Hazard ratios for relapse-free survival and overall survival in patients receiving adjuvant chemotherapy.

	RFS			OS		
Prognostic factors	HR	95% CI	P-value	HR	95% CI	P-value
ERCC1 level			0.026			0.031
<7.32	1			1		
≥7.32	2.16	1.09–4.25		2.21	1.07–4.55	
Staging			0.002			0.010
I, II and III	1			1		
IV	3.12	1.52–6.42		2.81	1.29–6.15	
Carcinoembryonic antigen (ng/ml)			0.012			0.050
≤5	1			1		
>5	2.49	1.23–5.09		2.16	0.99–4.68	

ERCC1, excision repair cross-complementation group 1; RFS, relapse-free survival; OS, overall survival; HR, hazard ratio; CI, confidence interval.
